# Novel anatomical apical dissection utilizing puboprostatic “open-collar” technique: Impact on apical surgical margin and early continence recovery

**DOI:** 10.1371/journal.pone.0249991

**Published:** 2021-04-15

**Authors:** Fumitaka Koga, Masaya Ito, Madoka Kataoka, Hiroshi Fukushima, Yasukazu Nakanishi, Kosuke Takemura, Hiroaki Suzuki, Kazumasa Sakamoto, Shuichiro Kobayashi, Ken-ichi Tobisu

**Affiliations:** Department of Urology, Tokyo Metropolitan Cancer and Infectious Diseases Center, Komagome Hospital, Tokyo, Japan; University Medical Center Utrecht, NETHERLANDS

## Abstract

**Purpose:**

To evaluate the impact of modifications to anatomical apical dissection including a puboprostatic open-collar technique, which visualizes the lateral aspect of the apex and dorsal vein complex (DVC) covering the rhabdosphincter while preserving the puboprostatic collar, on positive surgical margin (PSM) and continence recovery.

**Methods:**

One-hundred-and-sixty-seven patients underwent gasless single-port retroperitoneoscopic radical prostatectomy using a three-dimensional head-mounted display system. Sequentially modified surgical techniques comprised puboprostatic open-collar technique, sutureless transection of the DVC, retrograde urethral dissection, and anterior reconstruction. The associations of these modifications with PSM and continence recovery were assessed.

**Results:**

The puboprostatic open-collar technique, sutureless DVC transection, and retrograde urethral dissection were significantly associated with lower apical PSM (P = 0.003, 0.003, and 0.010, respectively). The former two also showed similar associations in 84 patients with anterior apical tumor (P = 0.021 and 0.030, respectively). Among 92 patients undergoing all of these three procedures, overall and apical PSM rates were 13.0% and 3.3%, respectively. Retrograde urethral dissection (odds ratio [OR] 2.73, P = 0.004) together with nerve sparing (OR 2.77, P = 0.003) and anterior apical tumor (OR 0.45, P = 0.017) were independently associated with immediate continence recovery. A multivariable model for 3-month continence recovery included anterior apical tumor (OR 0.28, P = 0.003) and puboprostatic open-collar technique (OR 3.42, P = 0.062). Immediate and 3-month continence recovery rates were 56.3% and 85.4%, respectively, in 103 patients undergoing both the puboprostatic open-collar technique and retrograde urethral dissection.

**Conclusion:**

Novel anatomical apical dissection utilizing a puboprostatic open-collar technique may favorably impact on both apical surgical margin and continence recovery.

## 1. Introduction

Apical dissection is one of the most challenging procedures in radical prostatectomy (RP) given the competing goals of cancer control and maintenance of urinary continence and sexual function. The apex represents the most common anatomical site of positive surgical margins (PSMs) [1]. Visualization of the apical structures and dissection with adequate surgical margins, particularly in cases with apical tumor, contribute to reduction of apical PSM in laparoscopic RP including robot-assisted RP [2, 3]. At the same time, preservation of the rhabdosphincter, functional urethral length, bladder neck, levator ani muscles and their fascia is required for maintenance of urinary continence [4–7].

We have adopted a three-dimensional head-mounted display (3D-HMD) system for single-port retroperitoneoscopic RP without using CO_2_ gas insufflation since October 2013. The general concept and techniques of gasless single-port retroperitoneoscopic RP using the 3D-HMD system (3D-RP) has been described in detail elsewhere [8–11]. Briefly, 3D-RP is carried out using a high-resolution stereovision in the wide extraperitoneal working space created along the anatomical plane through a single port of around 4 cm in diameter with affordable cost.

To improve cancer control and early continence recovery, we have modified the procedures for anatomical apical dissection in 3D-RP. The novelty of our modification is to better visualize lateral aspects of the apex and dorsal vein complex (DVC) covering the rhabdosphincter for better cancer control while preserving the structural continuity of the puboprostatic ligament (PPL) and arcus tendinous (AT), the so called “puboprostatic collar”[12, 13] for maintaining continence, which we term a “puboprostatic open-collar technique”. This procedure facilitates sutureless DVC transection using a bipolar sealing device distally beyond the anterior apical tumor, if present, combined with retrograde urethral dissection to secure apical resection margins while maximizing the functional urethral length.

In this study, we explored the impact of modified surgical techniques for anatomical apical dissection and pelvic floor reconstruction on surgical margin status and early continence recovery in patients who underwent 3D-RP.

## 2. Patients and methods

### 2.1. Surgical techniques

**Fig 1:**
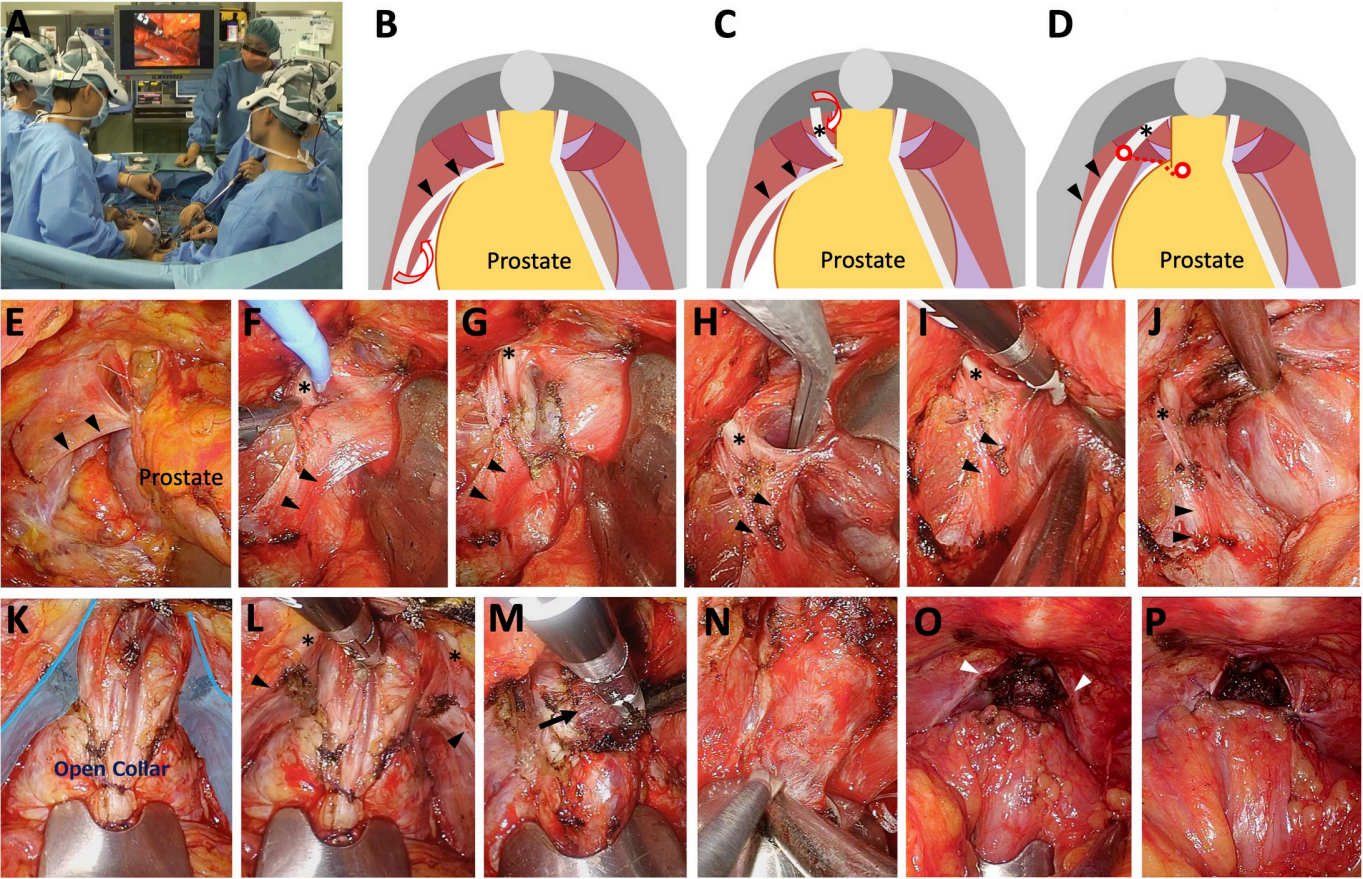
Surgical techniques for novel anatomical apical dissection and anterior reconstruction in gasless single-port retroperitoneoscopic radical prostatectomy. (A) The 3-dimensional head-mounted display system. (B-K) Puboprostatic open-collar technique. The surgical procedures consist of three steps: 1) Separating the endopelvic fascia (EF) lateral to the prostate (B), 2) separating the EF medial to the puboprostatic ligament (PPL) (C), and 3) transecting apical attachment of the pubococcygeus muscle (D). The EF is bluntly separated lateral to the prostate toward the apical attachment of the pubococcygeus muscle (B and E). Black arrowheads indicate preserved arcus tendinous (AT). Similarly, the EF is bluntly separated medial to the PPL, as indicated by an asterisk (C and F). The EF covering the apical attachment of the pubococcygeus muscle is incised to preserve the puboprostatic collar (D and G). Apical attachment of the pubococcygeus muscle (H) is transected using a bipolar sealing device (I). These procedures visualize the lateral aspect of the apical and urethral structures while preserving the puboprostatic collar (D and J). Open collar-shaped PPL-AT complexes (blue shadow) are preserved (K). (L and M) Sutureless transection of the dorsal vein complex (DVC). DVC is transected distally enough to secure apical surgical margins in cases of anterior apical tumor (L). The rhabdosphincter, indicated by an arrow, is exposed (M). (N) Retrograde urethral dissection. Overlying DVC tissues are retrogradely dissected from the rhabdosphincter and urethra toward the apex to secure apical surgical margins and to preserve the functional urethral length. The urethra is sharply divided. (O and P) Anterior reconstruction. Following vesico-urethral anastomosis, the detrusor muscle is anchored to the puboprostatic collar at its original apical attachment site (O) using a 3–0 Vicryl stich (P).

The 3D-HMD system comprises a 3D deflectable endoscope (Endoeye flex 3D deflectable videoscope, Olympus, Tokyo, Japan) and a 3D-HMD (Sony, Tokyo, Japan; Fig 1A). To create and maintain the surgical field, PLES retractors (Innomedics, Tokyo, Japan) and Omni-Tract pediatric retractors (Integra LifeSciences, Princeton, NJ) were used, respectively [8].

**Fig 2:**
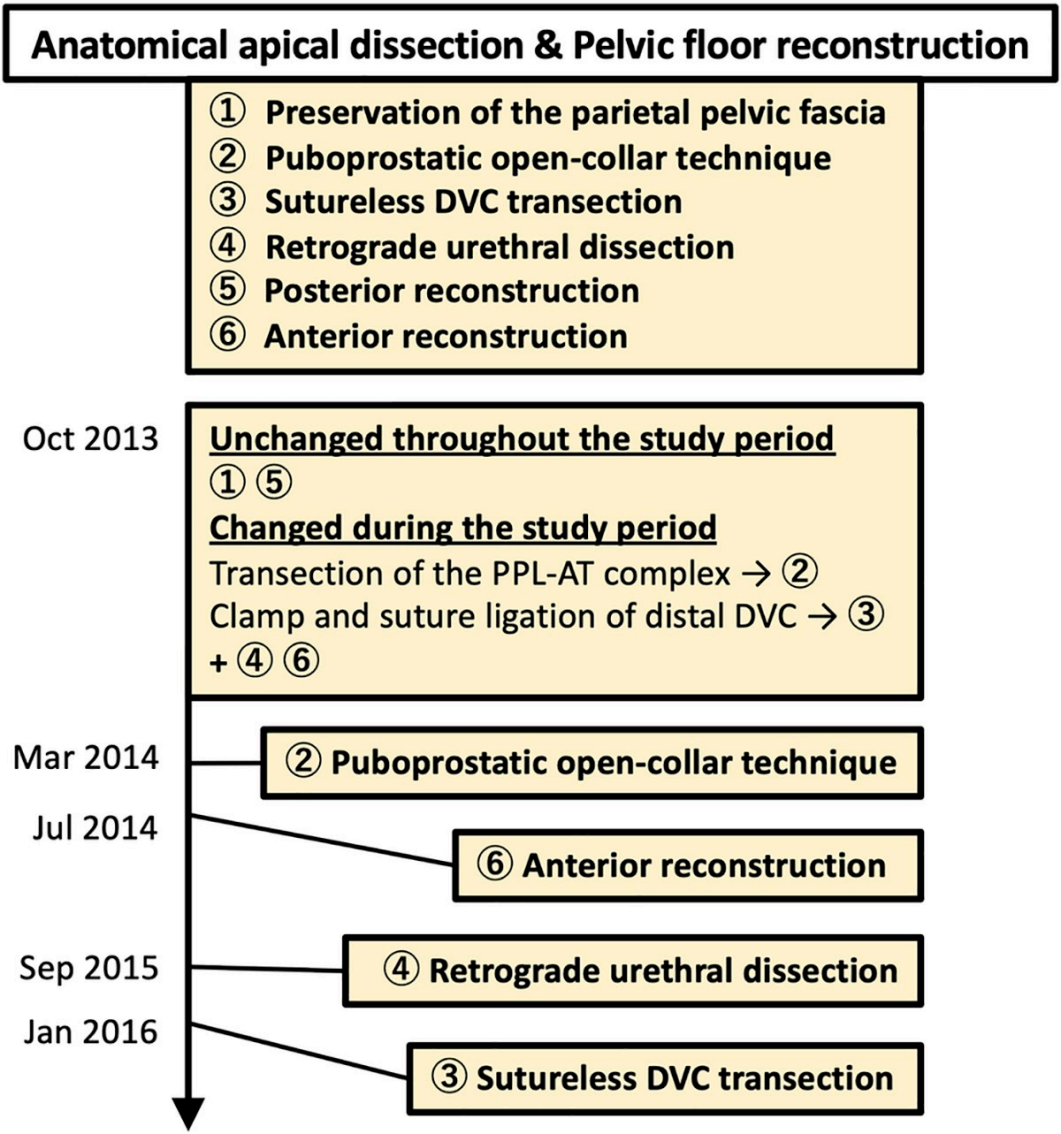
Modifications of anatomical apical dissection and pelvic floor reconstruction during the study period. Procedures ① and ⑤ remained unchanged throughout the study period. Transection of the puboprostatic ligament (PPL)-arcus tendinous (AT) complex, and distal clamp and suture ligation of the dorsal vein complex (DVC) were replaced by procedures ② and ③, respectively. Procedures ④ and ⑥ were newly introduced during the study period.

S1 Video shows points of surgical procedures. Fig 2 summarizes our surgical techniques for anatomical apical dissection, pelvic floor reconstruction, and their sequential modifications during the study period. The surgical techniques described in this study were implemented as a standard-of-care at our institution. Patients were assigned to each surgical procedure for anatomical apical dissection and pelvic floor reconstruction on or after time points indicated in Fig 2. 3D-RP is approved by the Ministry of Health, Labour and Welfare, and is covered by the Japanese universal health insurance system.

### 2.1.1 Patient positioning and preparation of a single port

Patients were placed in the temperate Trendelenburg position. The extraperitoneal pelvic space was developed via a suprapubic midline incision of around 5 cm and a single port was prepared using an Alexis wound retractor S (Applied Medical, Rancho Santa Margarita, CA).

#### 2.1.2 Pelvic Lymph Node Dissection (PLND)

Standard or extended PLND was performed when the percentage risk of lymph node involvement, estimated using the Briganti nomogram 2012, 2017, or 2018 [14–16], was 5–20% or >20%, respectively. A template of standard PLND includes inner external iliac, obturator, and outer internal iliac nodes. For extended PLND, the lymphatic chains of the external iliac artery, those medial to the internal iliac vessels, and distal common iliac area including Marcille’s fossa were removed in addition to the standard template. Fat tissues overlying the prostate were removed to expose the endopelvic fascia (EF) and PPL.

#### 2.1.3 Puboprostatic open-collar technique (Fig 1B–1K)

The lateral surface of the prostate was exposed unless intrafascial nerve sparing (NS) was attempted. Following the original technique of puboprostatic collar preservation [12, 13], the parietal pelvic fascia (PF) and visceral PF were bluntly separated antegradely toward the apex, where the PF were fused at the attachment site of the pubococcygeus muscle (Fig 1B and 1E). Similarly, the parietal and visceral PF were bluntly separated medial to the PPL in an attempt to preserve the parietal PF overlaying the pubococcygeus muscle (Fig 1C and 1F). Then, the EF covering apical attachment of the pubococcygeus muscle was sharply incised to preserve the puboprostatic collar (Fig 1D and 1G). Finally, apical attachment of the pubococcygeus muscle (Fig 1H) was transected using a bipolar sealing device (Fig 1I). Only the anterior part of the muscular attachment was released when intrafascial NS was attempted. These procedures visualized the lateral aspect of the apex and DVC covering the rhabdosphincter while preserving the puboprostatic collar (Fig 1J). After completing these procedures, open collar-shaped PPL-AT complexes were preserved (Fig 1K). Until March 2014, we used to transect the muscular attachment lateral to the PPL and thus the PPL-AT complex was divided (Fig 2).

#### 2.1.4 Sutureless transection of the DVC (Fig 1L and 1M)

The DVC was transected without suture ligation using a bipolar sealing device and scissors. The DVC was transected distally for adequate resection margins in cases of anterior apical tumor on MRI (Fig 1L), and the rhabdosphincter was exposed (Fig 1M). Until January 2016, DVC was clamped distally with long straight Pean forceps and suture-ligated, which had limited adequate resection margins in cases of anterior apical tumor (Fig 2).

#### 2.1.5 Dissection and reconstruction of the bladder neck

The bladder neck was incised and reconstructed in a tennis racket shape with mucosal eversion at three sites.

#### 2.1.6 Dissection of the vas deferens and seminal vesicles

The vas deferens were isolated and divided followed by seminal vesicle dissection.

#### 2.1.7 Lateral pedicle control

When planning intrafascial NS, the neurovascular bundle (NVB) was peeled off from the prostatic capsule. A plane was subsequently developed between the prostatic capsule and Denonvillier’s fascia posteriorly, proceeding distally and laterally. Otherwise, the Denonvillier’s fascia was separated or incised posteriorly to carry out interfascial/extrafascial NS or wide resection of the NVB.

#### 2.1.8 Retrograde urethral dissection (Fig 1N)

Overlying DVC tissues were retrogradely dissected from the rhabdosphincter and urethra toward the apex to secure apical margins and to preserve the functional urethral length (Fig 1N). Modest retrograde urethral dissection was attempted in cases of anterior apical tumor on MRI. The urethra was sharply divided and the prostate was freed. Until September 2015, the most proximal site of the urethra exposed after DVC transection was transected without retrograde urethral dissection (Fig 2).

#### 2.1.9 Pelvic floor reconstruction and anastomosis (Fig 1O and 1P)

Posterior reconstruction was done in two layers using continuous and mattress 3–0 Vicryl sutures. Following vesico-urethral anastomosis with six 3–0 Vicryl stitches, the detrusor muscle was anchored to the PPL-AT complex at its original apical attachment site using a 3–0 Vicryl stich (Fig 1O and 1P). Anterior reconstruction has been performed since July 2014.

### 2.2. Surgical outcomes

#### 2.2.1 Study population

A prospectively maintained RP database in which all data were fully anonymized was reviewed to retrieve the records of consecutive patients who underwent 3D-RP from October 2013 to June 2020 at a single cancer center. To maintain the database, patients’ medical records at Komagome Hospital were accessed between October 2013 and March 2021. Since the introduction of robotic RP in 2017, 3D-RP has been mainly performed for patients with locally advanced disease. Nine surgeons including six novices of <20 RP experiences performed 3D-RP, all of which were supervised by a senior surgeon (FK). All patients underwent mpMRI preoperatively. Risk classification was defined according to the European Association of Urology guidelines, in which clinical tumor stage (cT) 3–4 or clinical node stage (cN) 1 disease was classified as locally advanced disease [17]. Preoperative androgen deprivation therapy (ADT) was offered as a part of multimodal therapy to the majority of patients with locally advanced disease. A small subset of patients received preoperative ADT because of a change in treatment plan from radiation to RP. This study was approved by the Institutional Review Board of Komagome Hospital (#2400) and was conducted in accordance with the principles of the Declaration of Helsinki and Good Clinical Practice Guidelines. A written informed consent was obtained from all patients included in this study. The individuals in this manuscript (Fig 1 and S1 Video) have given written informed consent to publish these case details.

#### 2.2.2 Outcomes measurement and statistical analysis

Primary endpoints were overall PSM, apical PSM, continence recovery immediately (within a week) after catheter removal and at 3-months after RP. A urethral catheter was removed on postoperative day 5. Continence recovery was defined as no pad or 1 security liner per day by self-report, which was obtained at the outpatient visit at 1-month and 3-months after RP. Patients documented the time of pad exchange and weight of urine loss in bladder diary for 1-month after the catheter removal and the duration to achieve continence recovery was recorded in medical chart by reference to the bladder diary. Biochemical recurrence (BCR), defined as PSA elevation above 0.2 ng/mL, was also assessed.

Variables included in this study are listed in Table 1. Statistical analysis was performed using JMP software version 14 (SAS Institute, Cary, NC). The differences in frequency were evaluated using a chi-square test or Fisher’s exact probability test. Logistic regression analyses were used to evaluate variables associated with continence recovery. Cox proportional hazard model was used to assess variables associated with BCR. A reduced multivariable model was developed using the stepwise backward method, in which the variable with the highest P value was eliminated from each iteration of the multivariable analysis. A two-tailed P <0.05 was considered significant.

**Table 1:** Demographics of 167 men undergoing 3D-RP.

Variables	N (%)
Age (years)*	68 (45–78)
PSA (ng/mL)*	9.9 (1.9–271)
Clinical T stage	
T1c	6 (3.6)
T2a	79 (47.3)
T2b or 2c	36 (21.5)
T3a	28 (16.8)
T3b	15 (9.0)
T4	3 (1.8)
Clinical N stage	
N0	159 (95.2)
N1	8 (4.8)
Biopsy Gleason score	
3+3	14 (8.4)
3+4	28 (16.8)
4+3	41 (24.6)
8	52 (31.1)
9 or 10	32 (19.2)
Risk classification	
Low	2 (1.2)
Intermediate	65 (38.9)
High	53 (31.7)
Locally advanced	47 (28.1)
Anterior apical tumor	
Yes	84 (50.3)
No	83 (49.7)
Preoperative ADT	
No	115 (68.9)
Yes	52 (31.1)
Nerve-sparing surgery	
Intra- or interfascial	68 (40.7)
Extrafascial or none	99 (59.3)
Operation time (min)*	338 (233–457)
Blood loss (including urine, mL)*	580 (20–2548)
Blood transfusion	
No	163 (97.6)
Yes	4 (2.4)
Puboprostatic open-collar technique	
Yes	153 (91.6)
No	14 (8.4)
Anterior reconstruction	
Yes	144 (86.2)
No	23 (13.8)
Retrograde urethral dissection	
Yes	103 (61.7)
No	64 (38.3)
Sutureless DVC transection	
Yes	93 (55.7)
No	74 (44.3)
Pelvic lymph node dissection	
No	26 (15.6)
Standard	91 (54.5)
Extended	50 (29.9)
No. lymph nodes removed*	
Standard dissection	17 (2–50)
Extended dissection	30 (8–65)
Pathological T stage	
yT0	3 (1.8)
T2 or yT2	110 (66.9)
T3a or yT3a	32 (19.2)
T3b or yT3b	22 (13.2)
Pathological N stage	
Nx	26 (15.6)
N0	126 (75.4)
N1	15 (9.0)

*Median (range). 3D-RP, gasless single-port retroperitoneoscopic radical prostatectomy using the three-dimensional head-mounted display system. ADT, androgen deprivation therapy. DVC, dorsal vein complex.

## 3. Results

Table 1 summarizes the clinicopathological and operative demographics of 167 consecutive men included in the study. Median (range) age at the time of 3D-RP and PSA at the initiation of treatment was 68 years (45–78) and 9.9 ng/mL (1.9–271), respectively. The numbers of patients with low-risk, intermediate-risk, high-risk, and locally advanced disease were 2 (1.2%), 65 (38.9%), 53 (31.7%), and 47 (28.1%), respectively. Preoperative mpMRI showed anterior apical tumor in 84 (50.3%) patients. Preoperative ADT was given to 52 (31.1%) patients, of whom 38 (73.1%), 6 (11.5%), and 8 (15.4%) had locally advanced, high-risk, and intermediate-risk disease, respectively.

Puboprostatic open-collar technique, sutureless DVC transection, retrograde urethral dissection, and anterior reconstruction were performed in 153 (91.6%), 93 (55.7%), 103 (61.7%), and 144 (86.2%) patients, respectively. Standard and extended PLND was performed in 91 (54.5%) and 50 (29.9%) patients, respectively. Intra- or interfascial NS was performed in 68 (40.7%) patients. Median (range) operation time and blood loss including urine were 338 minutes (233–457) and 580 mL (20–2548), respectively. Intra- or post-operative blood transfusion was required in 4 (2.4%) patients. There was no significant association between the modified surgical techniques and operation time or blood loss. A surgical complication of Clavien-Dindo grade 3 or more was grade 3a deep venous thrombosis in a patient (0.6%).

Pathological (p) T stage was yT0, T2 or yT2, T3a or yT3a, and T3b or yT3b in 3 (1.8%), 110 (66.9%), 32 (19.2%), and 22 (13.2%), respectively. Fifteen (9.0%) patients had pN1.

### 3.1 Surgical margin outcomes

Overall and apical PSM were reported in 27 (16.2%) and 14 (8.4%) patients, respectively. The precise location of the apical PSM was as follows: the anterior apex alone in 9, the anterior and posterior apex in 2, the lateral apex in 2, and the posterior apex alone in 1. None had positive urethral margin. In cases of PSM, median (range) extent of PSM (a sum of PSM length, mm) was 4 (0.4–33). Higher pT stage (P <0.001) and pN1 (P = 0.030) were significantly associated with overall PSM (Table 2). Of note, apical PSM rates were significantly lower for a puboprostatic open-collar technique (P = 0.003), retrograde urethral dissection (P = 0.003), and sutureless DVC transection (P = 0.010) along with lower pT stage (P <0.001). Overall and apical PSM rates were 13.0% and 3.3%, respectively, in 92 patients who underwent all of the three modified techniques.

**Table 2:** Associations of clinicopathologic variables with surgical margin status.

Variables	Surgical margin, N (%)		*P* value	Apical surgical margin, N (%)		*P* value
	Positive	Negative		Positive	Negative	
Total	27 (16.2)	140 (83.8)		14 (8.4)	153 (91.6)	
Age (yeas)*	68 (45–78)	68 (54–76)	0.93	68 (45–78)	67 (59–74)	0.68
PSA (ng/mL)*	9.9 (1.9–271)	10.6 (5–192)	0.24	10.0 (1.9–271)	9.7 (5.0–28.0)	0.80
Clinical T stage			0.76			0.77
T1c or 2a	12 (14.1)	73 (85.9)		8 (9.4)	77 (90.6)	
T2b or 2c	4 (11.1)	32 (88.9)		1 (2.8)	35 (97.2)	
T3a	6 (21.4)	22 (78.6)		3 (10.7)	25 (89.3)	
T3b or 4	5 (27.8)	13 (72.2)		2 (11.1)	16 (88.9)	
Clinical N stage			1.00			1.00
N0	26 (16.4)	133 (83.7)		14 (8.8)	145 (91.2)	
N1	1 (12.5)	7 (87.5)		0 (0)	8 (100)	
Biopsy Gleason score			0.64			0.89
3+3	2 (14.3)	12 (85.7)		2 (14.3)	12 (85.7)	
3+4	4 (14.3)	24 (85.7)		3 (10.7)	25 (89.3)	
4+3	5 (12.2)	36 (87.8)		3 (7.3)	38 (92.7)	
8	8 (15.4)	44 (84.6)		4 (7.7)	48 (92.3)	
9 or 10	8 (25.0)	24 (75.0)		2 (6.3)	30 (93.8)	
Risk classification			0.34			0.77
Low or intermediate	7 (10.4)	60 (89.6)		7 (10.4)	60 (89.6)	
High	10 (18.9)	43 (81.1)		4 (7.6)	49 (92.5)	
Locally advanced	10 (21.3)	37 (78.7)		3 (6.4)	44 (93.6)	
Anterior apical tumor			0.40			0.16
Yes	16 (19.1)	68 (81.0)		10 (11.9)	79 (88.1)	
No	11 (13.3)	72 (86.8)		4 (4.8)	74 (95.2)	
Preoperative ADT			0.37			0.23
No	21 (18.3)	94 (81.7)		12 (10.4)	103 (89.6)	
Yes	6 (11.5)	46 (88.5)		2 (3.9)	50 (96.2)	
Nerve-sparing surgery			0.52			0.40
Intra- or interfascial	9 (13.2)	59 (86.8)		4 (5.9)	64 (94.1)	
Extrafascial or none	18 (18.2)	81 (81.8)		10 (10.1)	89 (89.9)	
Blood loss (including urine, mL)*	595 (90–2050)	550 (20–2548)	0.67	590 (110–1740)	550 (20–2548)	0.61
Puboprostatic open-collar technique			0.054			0.003
Yes	22 (14.4)	131 (85.6)		9 (5.9)	144 (94.1)	
No	5 (35.7)	9 (64.3)		5 (35.7)	9 (64.3)	
Retrograde urethral dissection			0.053			0.003
Yes	12 (11.7)	91 (88.4)		3 (2.9)	100 (97.1)	
No	15 (23.4)	49 (76.6)		11 (17.2)	53 (82.8)	
Sutureless DVC transection			0.41			0.010
Yes	13 (14.0)	80 (86.0)		3 (3.2)	90 (96.8)	
No	14 (18.9)	60 (81.1)		11 (14.9)	63 (85.1)	
Pathological T stage			<0.001			<0.001
yT0	0 (0)	3 (100)		0 (0)	3 (100)	
T2 or yT2	4 (3.6)	106 (96.4)		3 (2.7)	107 (97.3)	
T3a or yT3a	11 (34.4)	21 (65.6)		8 (25.0)	24 (75.0)	
T3b or yT3b	12 (54.6)	10 (45.5)		3 (13.6)	19 (86.4)	
Pathological N stage			0.030			0.95
Nx	3 (11.5)	23 (88.5)		2 (7.7)	24 (92.3)	
N0	18 (14.3)	108 (85.7)		11 (8.7)	115 (91.3)	
N1	6 (40.0)	9 (60.0)		1 (6.7)	14 (93.3)	

*Median (range). ADT, androgen deprivation therapy. DVC, dorsal vein complex.

Similar results were obtained in a subgroup of 115 ADT-naïve patients (Table 2); puboprostatic open-collar technique (P = 0.003), retrograde urethral dissection (P = 0.005), and sutureless DVC transection (P = 0.031) were significantly associated with negative apical surgical margins.

Among 84 patients with an anterior apical tumor, the puboprostatic open-collar technique (P = 0.021) and retrograde urethral dissection (P = 0.030) were significantly associated with negative apical surgical margins (Table 3). The former was also associated with a lower overall PSM rate (P = 0.045).

**Table 3:** Associations of clinicopathologic variables with surgical margin status in subsets of 115 ADT-naïve patients and 84 patients with anterior apical tumor on mpMRI.

Variables	ADT-naïve patients						Patients with anterior apical tumor					
	Surgical margin, N (%)		*P* value	Apical surgical margin, N (%)		*P* value	Surgical margin, N (%)		*P* value	Apical surgical margin, N (%)		*P* value
	Positive	Negative		Positive	Negative		Positive	Negative		Positive	Negative	
Total	21 (18.3)	94 (81.7)		12 (10.4)	103 (89.6)		16 (19.0)	68 (81.0)		10 (11.9)	74 (88.1)	
Anterior apical tumor			0.63			0.064						
Yes	11 (20.4)	43 (79.6)		9 (16.7)	45 (83.3)		16 (19.0)	68 (81.0)		10 (11.9)	74 (88.1)	
No	10 (16.4)	51 (83.6)		3 (4.9)	58 (95.1)		0	0		0	0	
Puboprostatic open-collar technique			0.028			0.003			0.045			0.021
Yes	16 (15.4)	88 (84.6)		7 (6.7)	97 (93.3)		13 (16.7)	65 (83.3)		7 (9.0)	71 (91.0)	
No	5 (45.5)	6 (54.6)		5 (45.5)	6 (54.6)		3 (50.0)	3 (50.0)		3 (50.0)	3 (50.0)	
Retrograde urethral dissection			0.23			0.005			0.057			0.030
Yes	9 (14.1)	55 (85.9)		2 (3.1)	62 (96.9)		7 (13.0)	47 (87.0)		3 (5.6)	51 (94.4)	
No	12 (23.5)	39 (76.5)		10 (19.6)	41 (80.4)		9 (30.0)	21 (70.0)		7 (23.3)	23 (76.7)	
Sutureless DVC transection			0.64			0.031			0.45			0.085
Yes	9 (16.4)	46 (83.6)		2 (3.6)	53 (96.4)		8 (16.3)	41 (83.7)		3 (6.1)	46 (93.9)	
No	12 (20.0)	48 (80.0)		10 (16.7)	50 (83.3)		8 (22.9)	27 (77.1)		7 (20.0)	28 (80.0)	
Pathological T stage			<0.001			0.003			<0.001			0.043
T2	3 (3.9)	73 (96.1)		3 (3.9)	73 (96.1)		4 (6.9)	54 (93.1)		3 (5.2)	55 (94.8)	
T3a	10 (40.0)	15 (60.0)		7 (28.0)	18 (72.0)		6 (33.3)	12 (66.7)		5 (27.8)	13 (72.2)	
T3b	8 (57.1)	6 (42.9)		2 (14.3)	12 (85.7)		6 (75.0)	2 (25.0)		2 (25.0)	6 (75.0)	

ADT, androgen deprivation therapy. mpMRI, multiparametric magnetic resonance image. ADT, androgen deprivation therapy. DVC, dorsal vein complex.

### 3.2 Continence outcomes

In total, immediate and 3-month continence recovery were achieved in 78 (46.7%) and 137 (82.0%) patients, respectively. In multivariable analysis (Table 4), anterior apical tumor was independently associated with adverse immediate and 3-month continence recovery (odds ratio [OR] 0.45, 95% confidence interval [CI] 0.23–0.87, P = 0.017; and OR 0.28, 95% CI 0.11–0.66, P = 0.003, respectively). NS was independently associated with favorable immediate continence recovery (OR 2.77, 95% CI 1.43–5.47, P = 0.003). Among surgical procedures modified, retrograde urethral dissection was independently associated with favorable immediate continence recovery (OR 2.73, 95% CI 1.37–5.57, P = 0.004). Puboprostatic open-collar technique remained in the final multivariable model for 3-month continence recovery but did not reach statistical significance (P = 0.062). Sutureless DVC transection was not associated with adverse continence recovery. Immediate and 3-month continence recovery rates were 56.3% and 85.4%, respectively, in 103 patients who underwent both puboprostatic open-collar technique and retrograde urethral dissection.

**Table 4:** Uni- and multivariable analysis for postoperative continence recovery.

Variables	Immediate recovery				3-month recovery			
	Univariable OR (95% CI)	*P* value	Multivariable OR (95% CI)	*P* value	Univariable OR (95% CI)	*P* value	Multivariable OR (95% CI)	*P* value
Age (years)	0.98 (0.93–1.03)	0.50			1.01 (0.95–1.09)	0.78		
PSA (ng/mL)	1.00 (0.99–1.01)	0.96			1.00 (0.99–1.01)	0.53		
Risk classification		0.15				0.70		
Low or intermediate	Reference				Reference			
High	0.75 (0.36–1.56)	0.45			0.69 (0.27–1.80)	0.45		
Locally advanced	0.57 (0.26–1.21)	0.14			0.77 (0.28–2.10)	0.60		
Anterior apical tumor		0.025		0.017		0.005		0.003
No	Reference		Reference		Reference		Reference	
Yes	0.49 (0.27–0.91)		0.45 (0.23–0.87)		0.30 (0.12–0.70)		0.28 (0.11–0.66)	
Preoperative ADT		0.67				0.55		
No	Reference				Reference			
Yes	0.87 (0.45–1.67)				1.30 (0.55–3.32)			
Nerve-sparing surgery		<0.001		0.003		0.18		
Intra- or interfascial	3.15 (1.67–6.05)		2.77 (1.43–5.47)		1.76 (0.77–4.32)			
Extrafascial or none	Reference		Reference		Reference			
Blood loss (including urine, mL)	1.00 (1.00–1.00)	0.22			1.00 (1.00–1.00)	0.31		
Puboprostatic open-collar technique		0.041				0.096		0.062
Yes	3.53 (1.05–16.0)				2.84 (0.82–8.98)		3.42 (0.93–11.7)	
No	Reference				Reference		Reference	
Anterior reconstruction		0.21				0.29		
Yes	1.77 (0.72–4.65)				1.76 (0.59–4.75)			
No	Reference				Reference			
Retrograde urethral dissection		0.001		0.004		0.15		
Yes	2.84 (1.49–5.55)		2.73 (1.37–5.57)		1.80 (0.81–4.01)			
No	Reference		Reference		Reference			
Sutureless DVC transection		0.040				0.77		
Yes	1.91 (1.03–3.58)				1.12 (0.50–2.48)			
No	Reference				Reference			
PLND		0.14				0.71		
No	Reference				Reference			
Standard	0.40 (0.16–0.99)	0.046			0.54 (0.12–1.81)	0.34		
Extended	0.42 (0.15–1.09)	0.075			0.51 (0.11–1.87)	0.32		
Pathological T stage		0.58				0.37		
yT0	0.53 (0.02–5.77)	0.61			7.32×10^5^ (0.19-∞)	0.32		
T2 or yT2	Reference				Reference			
T3a or yT3a	0.65 (0.28–1.43)	0.28			0.65 (0.25–1.84)	0.41		
T3b or yT3b	1.29 (0.51–3.30)	0.59			0.49 (0.17–1.51)	0.20		

ADT, androgen deprivation therapy. DVC, dorsal vein complex. PLND, pelvic lymph node dissection. OR, odds ratio. 95% CI, 95% confidence interval.

Among surgical procedures modified, retrograde urethral dissection was significantly associated with better immediate (P = 0.018) and 3-month (P = 0.041) continence recovery in the 84 patients with anterior apical tumor (S1 Table).

### 3.3 BCR

During followup (median 56 months, range 7 to 88), 46 patients experienced BCR and two patients died of the disease. The modified surgical techniques were not associated with BCR while extracapsular extension, seminal vesicle involvement, and intra- or interfascial NS were independently associated with BCR (S2 Table).

## 4. Discussion

Our modifications of anatomical apical dissection including the puboprostatic open-collar technique, retrograde urethral dissection, and sutureless DVC transection were associated with reduced apical PSM rates. In addition, the former two were associated with better continence recovery. Sutureless DVC transection using a bipolar sealing device was unlikely to compromise continence recovery even in cases of anterior apical tumor, an independent risk factor of persisting urinary incontinence. Novel anatomical apical dissection utilizing a puboprostatic open-collar technique may contribute to both reducing PSM and early continence recovery among men undergoing 3D-RP.

In the original techniques for puboprostatic collar preservation, the DVC is divided proximal to the PPL [12, 13]. In our puboprostatic open-collar technique, exposure of the lateral aspects of the apex and DVC covering the rhabdosphincter allows for transecting the DVC distally beyond the tumor in cases of anterior apical tumor, which potentially secures safer resection margins and may reduce apical PSM. At the same time, preservation of the parietal PF, including the puboprostatic collar, plays a pivotal role in maintenance of urinary continence because the levator ani muscles support actions of the rhabdosphincter in continence mechanisms [18] and autonomic nerve branches of pelvic plexus to the rhabdosphincter run underneath the parietal PF [19]. Indeed, our puboprostatic open-collar technique appears to have contributed to reducing apical PSM and improving 3-month continence recovery, although the latter did not reach a statistical significance in multivariable analysis. Because of the significant reduction in apical PSM while maintaining early continence recovery, the puboprostatic open-collar technique may be considered in cases with anterior apical tumor.

Retrograde urethral dissection and NS were associated with better immediate continence recovery, which corroborated the importance of preservation of the functional urethral length [5, 7, 20] and NS [21] in maintaining urinary continence. Of note, these surgical procedures were not associated with better 3-months continence recovery. Given that the puboprostatic open-collar technique was applied to all and most (64/68) cases of retrograde urethral dissection and NS, respectively, these surgical procedures may contribute to immediate continence recovery on the background of preserved fascial structures of the pelvic floor.

Retrograde urethral dissection was also associated with reduced apical PSM in the overall patient group and those with anterior apical tumor. More importantly, retrograde urethral dissection appears to have contributed to reducing apical PSM in cases with anterior apical tumor (Table 3), which may be ascribed to the puboprostatic open-collar technique that visualizes the apical anatomy and sutureless distal DVC transection in such cases. Because sutureless DVC transection did not worsen early continent recovery in patients with anterior apical tumor, thermal injury to the rhabdosphincter using a sealing device seemed to be minimal.

The presence of anterior apical tumor on MRI negatively influenced early continence recovery but not apical PSM. In cases of anterior apical tumor, apical dissection was performed with an attempt of more distal transection of the DVC and modest retrograde urethral dissection. Such individualized modifications of apical dissection may account for poorer continence recovery while maintaining a relatively low apical PSM rate in such cases.

Despite their associations with reduced PSM, our modified surgical techniques were not associated with reduced risk of BCR. Unexpectedly, NS but not PSM was independently associated with BCR. In our practice, intra- or interfascial NS was carried out unless MRI-positive lesions contacted the prostatic capsule even in patients with high-risk disease when they desired NS. Indeed, about a half of patients undergoing NS procedures had high-risk disease. Although NS did not compromise PSM rates (13% vs. 18% in non-NS patients) in our cohort, applying stricter criteria of NS might reduce the risk of BCR following NS procedures.

Several limitations exist in the present study. First, this study of a small patient cohort has possible selection bias due to the retrospective nature. Second, this study involved multiple surgeons including residents and lacked consideration of their learning curves in 3D-RP. Our surgical team had been familiar with gasless single-port retroperitoneoscopic RP using a conventional 2D endoscope. Because use of the 3D-HMD system facilitated surgical procedures compared with a 2D endoscope, we feel that learning curve effects were minimal for 3D-RP performed under supervision by experienced surgeons in terms of oncological and functional outcomes. Third, we did not evaluate the generalizability of our techniques in other minimally invasive modalities. In our experience, favorable oncological and functional outcomes have been obtained when applied to robotic RP. Reproducibility of our surgical techniques also needs to be evaluated. Fourth, we did not compare outcomes between the original puboprostatic collar preservation and open-collar technique. The original technique may yield better functional outcomes without compromising oncological outcomes in patients without apical tumor. Fifth, we did not preoperatively measure membranous urethral length, which reportedly influenced continence recovery [22]. Given that apical dissection was modified according to the tumor location, postoperative urethral length may also be important in our study cohort. Lastly, we did not assess postoperative sexual function.

## 5. Conclusions

Novel anatomical apical dissection utilizing a puboprostatic open-collar technique may favorably impact both the apical surgical margin and early continence recovery. These techniques warrant further studies to evaluate the generalizability and reproducibility of oncological and functional efficiency in minimally invasive RP other than 3D-RP.

## Supporting information

S1 Video Novel anatomical apical dissection including the puboprostatic open-collar technique, sutureless transection of the Dorsal Vein Complex (DVC), and retrograde urethral dissection. Surgical procedures are described in details.
(DOCX)

S1 Table Associations of surgical procedures with postoperative continence recovery in patients with anterior apical tumor. (DOCX)

S2 Table Uni- and multivariable analysis for biochemical failure. (DOCX)

S1 Data (XLSX)
