# Supplementary material for: Novel anatomical apical dissection utilizing puboprostatic “open-collar” technique: Impact on apical surgical margin and early continence recovery
Source: PLoS One. 2021 Apr 15;16(4):e0249991. doi: 10.1371/journal.pone.0249991 (PMC8049266; doi:10.1371/journal.pone.0249991)
Supplement: S1 Video — Surgical procedures are described in details. (DOCX) [file pone.0249991.s001.docx]

S1 Video: (doi:10.5061/dryad.2rbnzs7n9)

<https://datadryad.org/stash/share/BlUw1SX1eFy0DWWEgK0TVEoOofqJiC1-LRHSkYh5P1k>
