# Supplementary material for: Novel anatomical apical dissection utilizing puboprostatic “open-collar” technique: Impact on apical surgical margin and early continence recovery
Source: PLoS One. 2021 Apr 15;16(4):e0249991. doi: 10.1371/journal.pone.0249991 (PMC8049266; doi:10.1371/journal.pone.0249991)
Supplement: S1 Table — (DOCX) [file pone.0249991.s002.docx]

**S1 Table. Associations of surgical procedures with postoperative continence recovery in patients with anterior apical tumor**

| Variables | Immediate recovery | | *P* value | Recovery at 3M | | *P* value |
| --- | --- | --- | --- | --- | --- | --- |
|  | Yes | No |  | Yes | No |  |
| Total | 32 (38.1) | 52 (61.9) |  | 62 (73.8) | 22 (26.2) |  |
| Puboprostatic open-collar technique  Yes  No | 31 (39.7)  1 (16.7) | 47 (60.3)  5 (83.3) | 0.40 | 59 (75.6)  3 (50.0) | 19 (24.4)  3 (50.0) | 0.18 |
| Retrograde urethral dissection  Yes  No | 26 (48.2)  6 (20.0) | 28 (51.9)  24 (80.0) | 0.018 | 44 (81.5)  18 (60.0) | 10 (18.5)  12 (40.0) | 0.041 |
| Sutureless DVC transection  Yes  No | 23 (46.9)  9 (25.7) | 26 (53.1)  26 (74.3) | 0.068 | 38 (77.6)  24 (68.6) | 11 (22.5)  11 (31.4) | 0.45 |
| Anterior reconstruction  Yes  No | 30 (40.0)  2 (22.2) | 45 (60.0)  7 (77.8) | 0.47 | 57 (76.0)  5 (55.6) | 18 (24.0)  4 (44.4) | 0.23 |

DVC, dorsal vein complex.
