# Supplementary material for: Novel anatomical apical dissection utilizing puboprostatic “open-collar” technique: Impact on apical surgical margin and early continence recovery
Source: PLoS One. 2021 Apr 15;16(4):e0249991. doi: 10.1371/journal.pone.0249991 (PMC8049266; doi:10.1371/journal.pone.0249991)
Supplement: S2 Table — (DOCX) [file pone.0249991.s003.docx]

**S2 Table. Uni- and multivariable analysis for biochemical failure**

| Variables | Univariable HR (95% CI) | *P* value | Multivariable HR (95% CI) | *P* value |
| --- | --- | --- | --- | --- |
| Age (years) | 0.98 (0.93-1.03) | 0.32 |  |  |
| PSA (ng/mL) | 1.00 (0.99-1.01) | 0.52 |  |  |
| Risk classification  Low or intermediate  High  Locally advanced | Reference  1.42 (0.76-2.67)  1.26 (0.49-3.22) | 0.55  0.27  0.63 |  |  |
| Anterior apical tumor  No  Yes | Reference  0.61 (0.34-1.11) | 0.10 |  |  |
| Preoperative ADT  No  Yes | Reference  0.77 (0.39-1.51) | 0.44 |  |  |
| Nerve-sparing surgery  Intra- or interfascial  Extrafascial or none | 1.95 (1.09-3.49)  Reference | 0.024 | 2.69 (1.47-4.93)  Reference | 0.001 |
| Blood loss (including urine, mL) | 1.00 (1.00-1.00) | 0.42 |  |  |
| Puboprostatic open-collar technique  Yes  No | 0.80 (0.34-1.90)  Reference | 0.62 |  |  |
| Retrograde urethral dissection  Yes  No | 1.14 (0.62-2.09)  Reference | 0.67 |  |  |
| Sutureless DVC transection  Yes  No | 1.18 (0.64-2.18)  Reference | 0.60 |  |  |
| No. lymph nodes removed | 1.00 (0.98-1.02) | 0.91 |  |  |
| Pathological T stage  yT0  T2 or yT2  T3a or yT3a  T3b or yT3b | 1.78 (0.24-13.4)  Reference  2.98 (1.48-5.99)  5.75 (2.78-11.9) | <0.001  0.57  0.002  <0.001 | 1.34 (0.18-10.1)  Reference  3.66 (1.49-7.46)  7.12 (3.39-15.0) | <0.001  0.78  <0.001  <0.001 |
| Pathological N stage  N0 or Nx  N1 | Reference  4.51 (2.14-9.48) | <0.001 |  |  |
| PSM  No  Yes | Reference  2.55 (1.36-4.78) | 0.004 |  |  |
| Extent of PSM (mm) | 1.07 (1.02-1.12) | 0.004 |  |  |
| Highest Gleason score at PSM sites  3+3  3+4  4+3  8  9 or 10  x | Reference  0.58 (0.08-4.16)  1.36 (0.26-7.03)  NA  3.44 (0.55-21.8)  0.97 (0.13-7.22) | 0.54  0.59  0.72  0.19  0.98 |  |  |

ADT, androgen deprivation therapy. DVC, dorsal vein complex. PSM, positive surgical margin. HR, hazard ratio. 95%CI, 95% confidence interval, NA, not available.
